# Wearable hip-assist robot modulates cortical activation during gait in stroke patients: a functional near-infrared spectroscopy study

**DOI:** 10.1186/s12984-020-00777-0

**Published:** 2020-10-29

**Authors:** Su-Hyun Lee, Hwang-Jae Lee, Youngbo Shim, Won Hyuk Chang, Byung-Ok Choi, Gyu-Ha Ryu, Yun-Hee Kim

**Affiliations:** 1 Department of Physical and Rehabilitation Medicine, Center for Prevention and Rehabilitation, Heart Vascular Stroke Institute, Samsung Medical Center, Sungkyunkwan University School of Medicine, Irwon-ro 115, Gangnam-gu, Seoul, 06355 Republic of Korea; 2 Department of Health Sciences and Technology, Department of Medical Device Management and Research, Department of Digital Health, SAIHST, Sungkyunkwan University, Irwon-ro 81, Gangnam-gu, Seoul, 06351 Republic of Korea; 3 Samsung Research, Samsung Electronics, 56, Seongchon-gil, Seocho-gu, Seoul, 06756 Republic of Korea; 4 Department of Neurology, Neuroscience Center, Samsung Medical Center, Sungkyunkwan University School of Medicine, Irwon-ro 81, Gangnam-gu, Seoul, 06351 Republic of Korea; 5 Department of Medical Device Management and Research, SAIHST, Sungkyunkwan University School of Medicine, Irwon-ro 81, Gangnam-gu, Seoul, 06351 Republic of Korea; 6 The Office of R&D Strategy & Planning, Samsung Medical Center, Irwon-ro 81, Gangnam-gu, Seoul, 06351 Republic of Korea

**Keywords:** Wearable hip-assist robot, Stroke, Functional near infrared spectroscopy, Cortical activation

## Abstract

**Background:**

Gait dysfunction is common in post-stroke patients as a result of impairment in cerebral gait mechanism. Powered robotic exoskeletons are promising tools to maximize neural recovery by delivering repetitive walking practice.

**Objectives:**

The purpose of this study was to investigate the modulating effect of the Gait Enhancing and Motivating System-Hip (GEMS-H) on cortical activation during gait in patients with chronic stroke. Methods. Twenty chronic stroke patients performed treadmill walking at a self-selected speed either with assistance of GEMS-H (GEMS-H) or without assistance of GEMS-H (NoGEMS-H). Changes in oxygenated hemoglobin (oxyHb) concentration in the bilateral primary sensorimotor cortex (SMC), premotor cortices (PMC), supplemental motor areas (SMA), and prefrontal cortices (PFC) were recorded using functional near infrared spectroscopy.

**Results:**

Walking with the GEMS-H promoted symmetrical SMC activation, with more activation in the affected hemisphere than in NoGEMS-H conditions. GEMS-H also decreased oxyHb concentration in the late phase over the ipsilesional SMC and bilateral SMA (*P* < 0.05).

**Conclusions:**

The results of the present study reveal that the GEMS-H promoted more SMC activation and a balanced activation pattern that helped to restore gait function. Less activation in the late phase over SMC and SMA during gait with GEMS-H indicates that GEMS-H reduces the cortical participation of stroke gait by producing rhythmic hip flexion and extension movement and allows a more coordinate and efficient gait patterns.

*Trial registration* NCT03048968. Registered 06 Feb 2017

## Background

Stroke survivors can suffer several neurological impairments or deficits, such as hemiparesis, sensory and motor skills disorder, cognitive deficits, or disorders in communication and visuo-spatial perception. Hemiplegia is one of the most common impairments after stroke and significantly reduces walking ability. Poststroke hemiplegic gait is typically characterized by a reduced gait velocity and asymmetry of bilateral kinetic, kinematic and spatiotemporal parameters [1–3]. Gait function is an important factor in determining the ability to independently perform activities of daily living. Therefore, regaining gait ability is a primary goal in the rehabilitation program for stroke patients.

Robot-assisted therapy for gait rehabilitation after stroke is a potential and novel approach for facilitating the restoration of function and enhancing the neural recovery process after stroke. Advanced and intelligent robotic devices are able to provide high-intensity, high-dosage, and consistent training, while potentially reducing strain on therapists [4–6]. The relative merits of wearable versus stationary robots include potability and the ability to shift the location of treatment into a more real-world environment, including the home, community, and society. The Gait Enhancing and Motivating System-Hip (GEMS-H), developed by Samsung Electronics Co., Ltd. (Suwon, Republic of Korea), is a hip-type robotic exoskeleton. Our previous studies showed that GEMS-H improved gait function, muscle efficiency, and cardiopulmonary metabolic efficiency [7–10]. However, it is not yet known how GEMS-H assisted gait training modulates cortical activity of stroke patients.

Gait is mediated through complex neuronal networks in the central nervous system involving cortical, subcortical, and spinal regions [11]. Repetitive gait training may modify these networks and induce physiological plasticity to improve ambulation [12]. Assessment of cortical activation while the subject is moving is a key factor in promoting a better understanding of neural motor control. Currently, limited information is available on the cerebral mechanisms underlying locomotor recovery after stroke because of technical limitations in assessing cerebral activation during execution of motor tasks. To date, various non-invasive methods have been used to acquire brain signals, including functional magnetic resonance imaging (fMRI), electroencephalography (EEG), positron emission tomography (PET), and functional near-infrared spectroscopy (fNIRS). fNIRS is a relatively new optical neuroimaging technique that enables visualization of cortical activation during human gait [13]. Although fNIRS has limited depth sensitivity that restricts the measurements of brain activity to cortical layers [14], this technique allows the noninvasive measurement of cortical activity with relatively good spatial and temporal resolution [15]. Compared to other neuroimaging devices, its advantages such as less sensitive to motion artifacts, cheap, portable, safe, and silent, [16] make it the choice for comprehensive and promising results in examination of stroke patients during rehabilitation [13, 17–20].

In this study, we aimed to identify how the wearable hip-assist robot modulates cortical activation during gait in patients with stroke. We hypothesized that GEMS-H-assisted walking would induce better automatic control of gait compared with walking without assistance of GEMS-H (NoGEMS-H), expressed as a reduction in cortical activation compared with the NoGEMS-H conditions. We also speculated that assistance with GEMS-H would lead to a more symmetrical cortical activation compared with NoGEMS-H conditions.

## Methods

### Participants and initial screening

A total of 20 people over a 3-months period after a unilateral stroke were included in this study; the characteristics of these subjects are shown in Table 1. Participants had to be able to stand and walk independently or under supervision (Functional Ambulation Categories, range, 3 to 5). Based on a clinical assessment, we excluded individuals with a history of other neurological disorders (except stroke) and musculoskeletal disorders that affected walking capacity, efficiency, and endurance. Written informed consent was obtained from all participants before entering the study. The study procedures were approved by the ethics committee of the Samsung Medical Center Institutional Review Board (Approval Number: 2016-07-093).

**Table 1:** Characteristics of participants

Characteristics	Values
Sex (male/female)	13/7
Age, years	61.74 (6.93)
Height, cm	164.37 (7.06)
Weight, kg	66 (9.12)
Stroke onset duration, month	36.67 (26.61)
Handedness (right/left)	20/0
Etiology	
Ischemic/hemorrhagic	6/14
Stroke location	
Cortical/subcortical/mixed	1/10/9
Side of stroke	
Right/left	12/8
Functional ambulation categorical	4.2 (0.83)
Berg balance scale	38.5 (5.65)

Values are expressed as mean (SD)

### Wearable hip-assist robot, GEMS-H

**Fig. 1:**
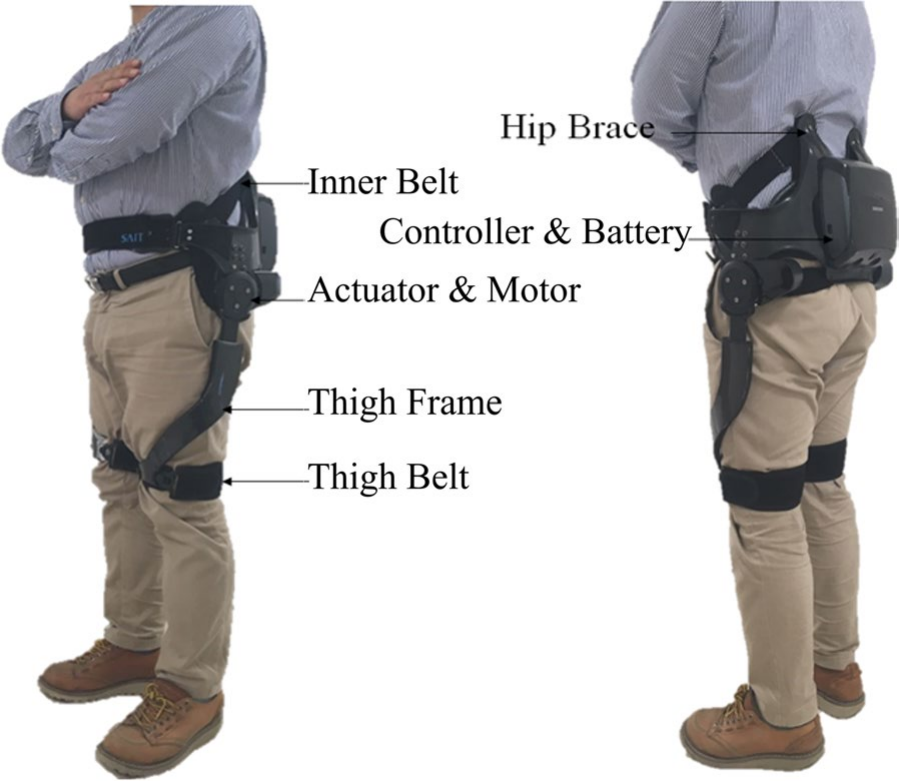
Gait Enhancing and Motivating System-Hip (GEMS-H)

The GEMS-H was developed at the Samsung Advanced Institute of Technology (Samsung Electronics Co., Ltd., Korea) as a wearable Hip-assist Robot with an assist-as-needed algorithm for stroke patients with gait disorder. This robot was designed to deliver active-assistance torque to the both hip or hip joint of the paretic side for extension and flexion. The GEMS-H has a lightweight (2.8 kg), comfortable and slim design that can be adjusted to fit the user’s body (Fig. 1). For more information regarding the strategy of assistive algorithms used for the GEMS-H, please see our previous paper [10].

### Experimental design and equipment

**Fig. 2:**
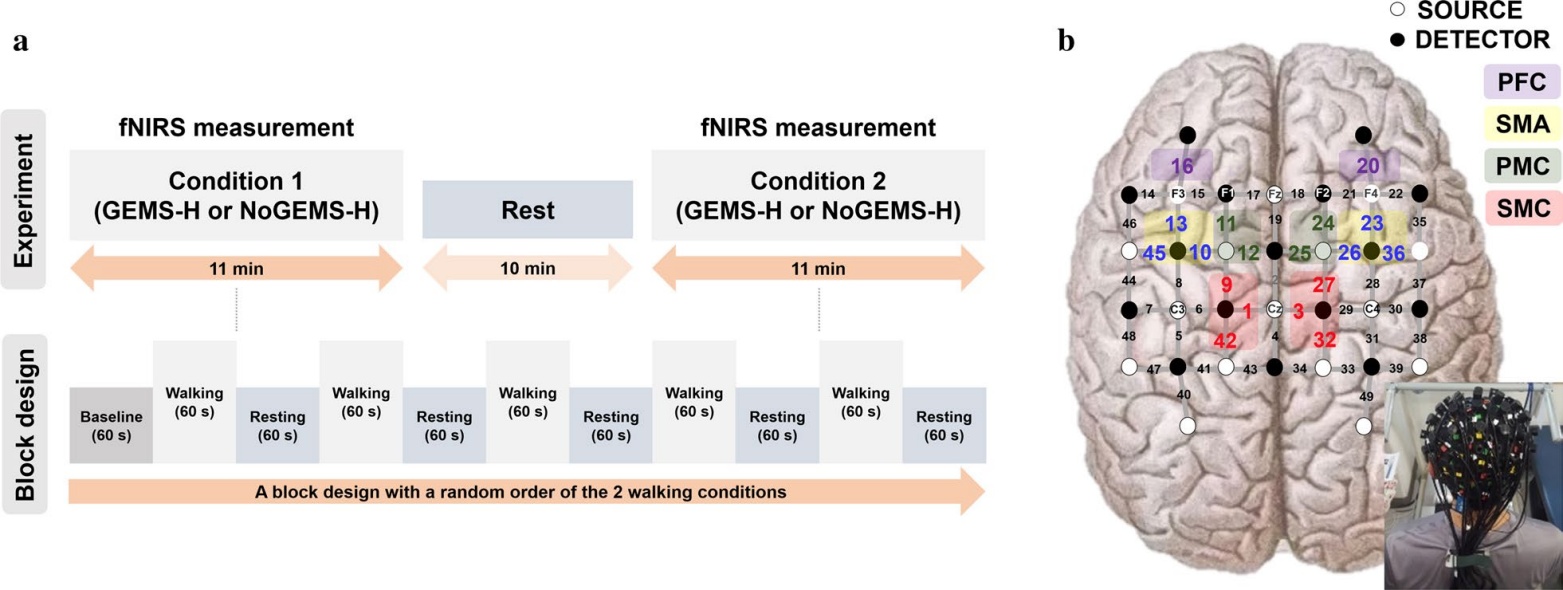
**a** Experimental design: the block design time course, including baseline, walking, and resting periods. **b** Location of optodes. The fNIRS system consists of 16 light source (white) and 16 detector (black) fibers, resulting in a total of 49 channels distributed over left SMC (Channels 1, 9, 42), right SMC (Channels 3, 27, 32), left SMA (Channels 11, 12), right SMA (Channels 24, 25), left PMC (Channels 10, 13, 45), right PMC (Channels 23, 26, 36), left PFC (Channel 16), and right PFC (Channel 20). *fNIRS* functional near infrared spectroscopy, *SMC* primary sensorimotor cortex, *SMA* supplemental motor areas, *PMC* premotor cortex, *PFC* prefrontal cortex, *GEMS-H* walking with assistance of GEMS-H, *NoGEMS-H* walking without assistance of GEMS-H

This study was designed as a crossover randomized controlled trial. All participants completed a familiarization session by walking on a treadmill and their preferred walking speed was recorded. None of the participants had difficulty walking on the treadmill during this initial walk. All participants were further acclimated to the GEMS-H through a single training session of 30 min with a licensed physical therapist. For fNIRS recording, participants were assigned to two consecutive tasks: a treadmill walking task at a self-selected speed (*a*) with the assistance of the GEMS-H (GEMS-H) or (*b*) without GEMS-H (NoGEMS-H) assistance. The experiment began with a fixed standing condition (60 s), followed by one of the two walking conditions (60 s) and then a resting condition (60 s) for five repetitions (block design) (see Fig. 2a). All participants were given specific instructions not to talk or laugh during testing and the participants rested by sitting for 10 min between the two tasks [21].

An fNIRS imaging system (NIRSscout® system, NIRx Medical Technology, Berlin, Germany) with two different wavelengths of 760 and 850 nm was used to record changes in oxygenated hemoglobin (oxyHb) concentration. The fNIRS optodes consisted of 16 LED light sources and 16 detectors, and a total of 49 useful source-detector channels were used for monitoring the hemodynamics of the bilateral primary sensorimotor cortex (SMC), premotor cortices (PMC), supplemental motor areas (SMA), and prefrontal cortices (PFC). The cranial vertex (Cz) located beneath the 1st source was the marker for ensuring replicable placement of the optodes. After the Cz position was determined on the participant’s head, an fNIRS head cap was placed on the participant’s head. The fNIRS head cap was designed to be compatible with the International 10–20 system and the interoptode distance was 3.0 cm. The fNIRS data were continuously acquired at a sample rate of 3.91 Hz through NIRStar Software version 14.2 (NIRx Medical Technologies LLC, Berlin, Germany) in MATLAB (The Mathworks, USA), which allowed oxyHb signals to be visualized in real time during data collection.

### Data preprocessing and analysis

Changes in oxyHb concentration during two different tasks (GEMS-H and NoGEMS-H) were analyzed by the nirsLAB® software version 2017.06 (NIRx Medical Technologies LLC, Berlin, Germany) in MATLAB. Cortical regions assessed included SMC (Brodmann area 1, 2, 3, and 4, medial), PMC (Brodmann area 6, lateral), SMA (Brodmann area 6, medial), and PFC (Brodmann area 9). We used oxyHb concentration as a marker for cortical activation because oxyHb is more sensitive indicator of brain activity during human locomotion-related activities than deoxygenated hemoglobin (deoxyHb), and there was a task-related increase of oxyHb concentration in the SMC without significant changes in deoxyHb concentration [19, 22]. OxyHb has been shown to have a higher signal to noise ratio associated with scattering of light through the scalp, skull, and inactive brain tissue [23]. For easy comparison, brains of patients are left–right flipped in the data preprocessing stage so that the stroke lesion of each subject were localized to the right hemisphere.

The fNIRS data were preprocessed to delete experimentally irrelevant time intervals from data, to remove motion artifacts, and to apply bandpass frequency filter to exclude experimentally irrelevant frequency bands. Using the components of Data Preprocessing available in nirsLAB®, discontinuities and spike artifacts of signals obtained from the 49 channels were removed and replaced by the nearest signals. The fNIRS signals were bandpass-filtered (low-cutoff frequency 0.01 Hz and high-cutoff frequency 0.2 Hz) to eliminate the effects of heartbeat, breathing, and low-frequency signal drifts for each wavelength [21]. The acquired fNIRS signal can contain various noises that can be classified as experimental errors, instrument noise, and physiological noise. The experimental errors and instrumental noise are not related to the brain activities, so they were eliminated prior to converting the raw optical density signals into a change in oxyHb concentration, and the preprocessed signals were then converted to relative concentration changes in oxyHb using the modified Beer-Lambert law for each source-detector channel [23, 24]. Finally, the oxyHb concentration changes were averaged over 5 repetitions for each walking condition to improve the signal-to-noise ratio [25].

From the processed fNIRS signals, oxyHb concentration was averaged per region of interest (ROI) (i.e., bilateral SMC, PMC, SMA, and PFC) [26]. The SMC was assessed with the medial parts of the posterior channels (channels 1, 9, and 42 in the left hemisphere and 3, 27, and 32 in the right), the SMA was assessed with the medial parts of the middle channels (channels 11 and 12 in the left hemisphere and channels 24 and 25 in the right), the PMC was assessed with the lateral parts of the middle channels (channels 10, 13, 45 in the left hemisphere and channels 23, 26, 36 in the right) and the PFC was partially assessed with channel 16 in the left hemisphere and channel 20 in the right hemisphere (see Fig. 2b) [19, 22, 27]. In this study, to analyze cortical activation, task periods were divided into an early and late phase. The period between 1 and 30 s of the task was defined as the early phase to reflect the immediate hemodynamic response for walking. The period between 31 and 60 s of the task was defined as the late phase to reflect continuous brain activity during walking as Lu et al. [21] described in the previous study. The initial and final 5 s of each task period were excluded to eliminate the transient periods between hemodynamic responses [28]. Block designs with a task period of 20–30 s are commonly used for fNIRS studies [29–31], but in this study, a longer task period (60 s) was used to investigate cortical activation. For quantification of activation between the serial measurements in two different tasks, we calculated ΔoxyHb in each channel, defined as oxyHb during Task Period – oxyHb during Rest Period.

### Statistical analysis

All statistical analyses were performed with SPSS version 22.0 (IBM, Armonk, NY, USA), and the significance level was set at 0.05. Descriptive statistics are expressed as mean ± standard deviation (SD) of the mean. Brain activation during each walking condition and phase was identified as a significant increase in oxyHb concentration by performing independent *t*-tests with false discovery rate (FDR) correction of multiple comparison for 49 channels. Within each walking condition, paired *t*-tests were used to compare activation in ipsi- and contralesional hemispheres.

## Results

### Patterns of cortical activation in different walking conditions

The mean values of oxyHb concentration during each walking condition and phase are presented in Table 2. OxyHb concentration over bilateral SMC in the early phase of gait was significantly higher in the GEMS-H than the NoGEMS-H condition (*P* < 0.05). In addition, we observed lower oxyHb concentration over the ipsilesional SMC and bilateral SMA in the late phase of gait (*P* < 0.05). In the bilateral PFC, oxyHb concentration in the late phase under GEMS-H condition was significantly higher than that of the NoGEMS-H condition (*P* < 0.05).

**Table 2:** The mean values of oxygenated hemoglobin concentration during each walking condition and phase

(Unit: mol*10^–3^)	GEMS-H	NoGEMS-H	*P*-value
Ipsilesional SMC			
Early phase	0.29 (0.22)	0.14 (0.19)	0.028*
Late phase	− 0.22 (0.14)	− 0.11 (0.10)	0.008**
Contralesional SMC			
Early phase	0.21 (0.15)	0.11 (0.16)	0.048^*^
Late phase	− 0.14 (0.09)	− 0.08 (0.09)	0.066
Ipsilesional SMA			
Early phase	0.26 (0.19)	0.17 (0.16)	0.095
Late phase	− 0.22 (0.10)	− 0.14 (0.13)	0.027*
Contralesional SMA			
Early phase	0.23 (0.17)	0.18 (0.15)	0.288
Late phase	− 0.20 (0.12)	− 0.08 (0.11)	0.003**
Ipsilesional PMC			
Early phase	0.20 (0.13)	0.21 (0.17)	0.692
Late phase	− 0.12 (0.12)	− 0.08 (0.13)	0.334
Contralesional PMC			
Early phase	0.16 (0.18)	0.25 (0.25)	0.200
Late phase	− 0.11 (0.07)	− 0.08 (0.08)	0.203
Ipsilesional PFC			
Early phase	0.13 (0.08)	0.17 (0.09)	0.156
Late phase	− 0.05 (0.06)	− 0.12 (0.06)	0.001**
Contralesional PFC			
Early phase	0.11 (0.14)	0.19 (0.16)	0.083
Late phase	− 0.03 (0.10)	− 0.11 (0.08)	0.005**

Values are presented as mean (SD)

^*^P < 0.05; **P < 0.01

*SMC* primary sensorimotor cortex, *SMA* supplemental motor areas, *PMC* premotor cortex, *PFC* prefrontal cortex, *GEMS-H* walking with assistance of GEMS-H, *NoGEMS-H* walking without assistance of GEMS-H

### Results of oxyhemoglobin concentration changes in time-series analysis

**Fig. 3:**
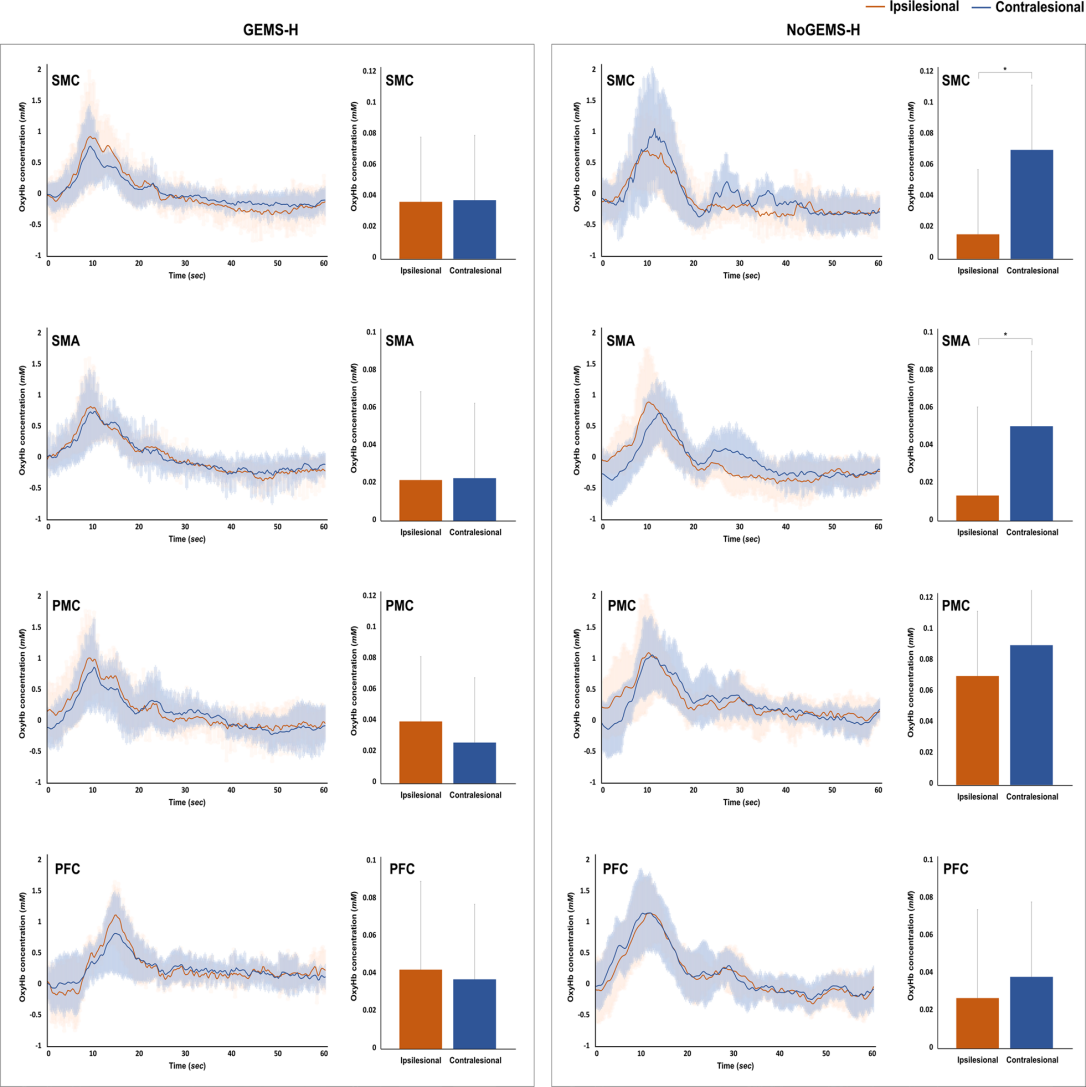
The average group changes in oxyHb concentrations in each ROI under GEMS-H and NoGEMS-H conditions. *P < 0.05. SMC, primary sensorimotor cortex; SMA, supplemental motor areas; PMC, premotor cortex; PFC, prefrontal cortex; GEMS-H, walking with assistance of GEMS-H; NoGEMS-H, walking without assistance of GEMS-H

The average changes of oxyHb concentrations in each ROI under GEMS-H and NoGEMS-H conditions are shown in Fig. 3. The hemodynamic responses from 5 to 55 s after task onset was analyzed to eliminate the transient periods between task conditions. In the NoGEMS-H condition, there was a statistically significant difference in oxyHb concentration between ipsi- and contralesional hemispheres in SMC and SMA (*P* < 0.05). In the GEMS-H condition, there was no statistically significant difference in oxyHb concentration between ipsi- and contralesional hemispheres at all ROIs.

## Discussion

The present study investigated the modulating effect of the wearable hip-assist robot, GEMS-H on cortical activation during gait in patients with chronic stroke. The main findings were that walking with GEMS-H promoted symmetrical SMC activation, with more activation in the affected hemisphere than the NoGEMS-H condition. Also, the GEMS-H decreased oxyHb concentration in the late phase over the ipsilesional SMC and bilateral SMA. In addition, in the bilateral PFC, oxyHb concentration of late phase under GEMS-H condition was significantly higher than that of the NoGEMS-H condition.

After stroke, brain neurophysiology and organization are changed resulting in brain-activation patterns that are different from those of healthy individuals [17]. Major differences in cortical activation pattern between normal and hemiparetic gait are asymmetrical primary SMC activation, with less activation in the affected hemisphere than in the unaffected hemisphere and recruitment of other motor-related areas such as the PMC and pre-supplementary motor area (pre-SMA) [19, 28]. In this study, we showed that walking with GEMS-H resulted in a more symmetrical SMC activation pattern, with higher SMC activation in the affected hemisphere than in the NoGEMS-H condition. In patients with stroke, asymmetrical brain activation is associated with hemiparetic gait. Improvement in the asymmetry of SMC activation has a significant correlation with the improvement in gait asymmetry. This suggests that symmetrical SMC activation may play an important role in restoring locomotor function after stroke [19]. These results indicate that GEMS-H promoted more SMC activation and a balanced activation pattern that helped to restore gait function in patients with stroke.

Gait is considered an automated, over-learned, and rhythmic motor task [32]. Humans possess a central pattern generator (CPG) to facilitate rhythmic and repetitive locomotor patterns via supraspinal regulation of cerebral neural networks [33]. Specific damage in supraspinal structures results in specific alterations in human locomotion, as evident in subjects with brain injuries such as stroke [34]. Our results revealed reduced oxyHb concentration over the ipsilesional SMC and bilateral SMA in the late phase of gait with the GEMS-H compared with the NoGEMS-H condition. Increased activity in SMC and SMA in the late phase during walking without the GEMS-H may indicate a compensatory activation in stroke patients due to impaired brain function and loss of automatism. Our previous study [10] demonstrated that the GEMS-H enhanced symmetricity of gait in stroke patients. Less activation in the SMC and SMA in the late phase during gait with GEMS-H might be a reflection of more symmetric and coordinated gait patterns by assisting rhythmic hip flexion and extension movements in gait with the GEMS-H. Increased gait symmetricity accompanied with reduced cortical participation in stroke gait made us assuming that GEMS-H facilitates the role of CPG, the subcortical neural substrate of gait, and contributes for automatic and symmetric gait patterns. This possibility, of course, warrants clarification by future studies.

Compared with NoGEMS-H conditions, walking with the GEMS-H elicited greater prefrontal activities in the late phase. This increased oxyHb concentration is thought to demonstrate a rise in aerobic metabolism in the PFC, which is a reflection of increased cellular activity [35, 36]. Increased attention to learn walking with the GEMS-H may promote PFC neuronal activity. Previous studies demonstrated that attentional control to engage specific motor tasks initially involves the prefrontal–parietal pathway followed by increased prefrontal activity. With continuous gait training, this activity attenuates as the striatal–cerebellum pathway assumes the neuronal process with increased automaticity [37, 38]. In our study, during walking with the GEMS-H, participants might have used more attention to learn walking with the GEMS-H and thus showed higher prefrontal activity in the late phase.

To our knowledge, this is the first study to investigate the modulating effect of the wearable hip-assist robot on cortical activation during gait in patients with chronic stroke. Our findings may provide evidence that gait with the GEMS-H increases the efficiency of cortical neural resources during walking by redistributing cortical components of gait function. However, there are some limitations to this study. First, we only demonstrated the temporary effect of the GEMS-H. Modulating effects on cortical-subcortical activities after long-term training with GEMS-H need to be conducted in the future using different functional imaging modalities such as fMRI or MEG. Second, there was a potential lack of statistical power due to the small sample size, therefore, our results cannot be generalized to the entire stroke population. Future research should be performed using a larger sample size.

## Conclusions

The present study shows the modulating effect of GEMS-H on cortical activation during gait in patients with chronic stroke. The results of the present study reveal that the GEMS-H promoted more SMC activation and a balanced activation pattern that helped to restore gait function. Less activation in the late phase over SMC and SMA during gait with GEMS-H indicates that GEMS-H reduces the cortical participation of stroke gait by producing rhythmic hip flexion and extension movement. These results suggest that the GEMS-H may be useful to allow a more coordinate and efficient gait patterns for stroke patients.

## Data Availability

The data sets supporting the conclusions of this article are included within the manuscript.

## Abbreviations

fNIRS: Functional near infrared spectroscopy
SMC: Primary sensorimotor cortex
SMA: Supplemental motor areas
PMC: Premotor cortex
PFC: Prefrontal cortex
GEMS-H: Walking with assistance of GEMS-H
NoGEMS-H: Walking without assistance of GEMS-H
oxyHb: Oxygenated hemoglobin
deoxyHb: Deoxygenated hemoglobin
